# National Medical Convention

**Published:** 1846-06

**Authors:** 


We had expected a report of the proceedings of the National Medical Convention, from a delegate in our own vicinity, but as we have not received it in time for publication, we insert the following from the *Boston Medical and Surgical*

*Journal* of May 13; thus renewing our obligations to its efficient editor, who is never backward in communicating to the profession, early and interesting intelligence. W. B. H.

*National Medical Convention.*—The delegates to this Convention met at the Medical College of the University of New York on Tuesday of last week. At the preliminary organization, Dr. Bell, of Philadelphia, was Chairman, and Dr. Buel, of New York, Secretary. The committee appointed to examine the credentials of the delegates, reported that all accredited delegates from any regularly organized society, local and voluntary associations as well as regular colleges, institutions and societies, be considered members of the convention, which report was accepted. Sixteen States were found to be represented (by delegates from State or other societies), and a committee of one from each State was appointed to nominate officers of the Convention, who presented the following nominations, which were unanimously confirmed, viz.: for President, Dr. J. Knight, of New Haven, Conn.; for Vice Presidents, Dr. Edward Delafield, of New York City, and Dr. John Bell, of Philadelphia; for Secretaries, Dr. Arnold, of Savannah, Geo., and Dr. Stille, of Philadelphia. Dr. G. S. Bedford, representing the University of New York, then moved that whereas the original object of the Convention, that of a *National* representation, for the good of the profession, had been defeated by the non-representation of many of the States, and most of the Medical Colleges and Societies, the Convention adjourn *sine die*. This motion was seconded by Dr. Paterson, also of the New York University. The vote was taken individually, and not by States, and was decided by yeas, 2; nays, 74. On account of this motion, Dr. Clymer, of Philadelphia, moved that the future sittings of the Convention be held elsewhere than at the University College; and another member proposed an amendment, that an adjournment immediately be made to the College of Physicians and Surgeons. Drs. Bedford and Paterson disclaimed all intention of opposing the Convention, and it was decided that Dr. Clymer's motion be laid on the table. A committee of nine was appointed to bring the subject of Medical Education before the Convention, consisting of Drs. Davis, March, Hayes, Walter, Bush, Bell, Haxhall, and the President.

The accredited delegates present on Tuesday were from the following institutions:—Vermont—Castleton Medical College, Vermont Medical College; N. Hampshire—Centre District Medical Society; Connecticut—State Medical Society and Medical Institution of Yale College; New York—State Medical Society, Medical Society of City and County, Bloomingdale Asylum, College of Physicians and Surgeons, King's

Co. Medical Society, University of the City of New York, Buffalo Medical Association, Erie Co. Medical Society, Albany Medical College, Geneva Co. Medical Society, Geneva Medical College, Madison Co. Medical Society, New York Hospital; Pennsylvania—Philadelphia Medical Society, Pennsylvania College; New Jersey—private individuals; Delaware—State Medical Society, Medical Association of Wilmington; Maryland—Medical College of Baltimore; Virginia—State Medical Society; Georgia—State Medical Society; Mississippi—State Medical Society; Indiana—La Porte University; Illinois—Medical Department of Illinois College; Tennessee—State Medical Society; Rhode Island—State Medical Society. And on Wednesday, the State Medical Societies of Vermont and Missouri were represented, also the Lunatic Asylum of Hudson and the New York Lunatic Asylum.

The following resolutions were presented on Wednesday by Dr. Davis, of the Committee on Medical Eeducation, and after discussion were unanimously adopted:—

"*Whereas*, it has been shown by experience that the association of persons engaged in the same pursuit, facilitates the attainment of their common objects; therefore,

"1st. *Resolved*, That it is expedient for the Medical Profession of the United States, to institute a *National Medical Association*, for the protection of their interests, for the maintenance of their honor and respectability, for the advancement of their knowledge, and the extension of their usefulness.

"2d. *Resolved*, That a Committee of seven be appointed to report a plan of organization for such an association, at the meeting to be held in Philadelphia, on the first Wednesday in May, 1847.

"3d. *Resolved*, That a Committee of seven be appointed to prepare and issue an Address to the different regularly organized Medical Societies, and chartered Medical Schools, in the United States, setting forth the objects of the National Medical Association, and inviting them to send delegates to a Convention, to be held in Philadelphia on the first Wednesday in May, 1847.

"4th. *Resolved*, That it is desirable that a uniform and elevated standard of requirements for the degree of 'M.D.' should be adopted by all the Medical Schools in the United States, and that a Committee of seven be appointed to report on this subject, at the meeting to be held in Philadelphia, on the first Wednesday in May, 1847.

"5th. *Resolved*, That it is desirable that young men, before being received as students of medicine, should have acquired a suitable preliminary education, and that a Committee of seven be appointed to report on the standard of acquirements, which should be exacted of such young men, and to report at the meeting, to be held on the first Wednesday in May, 1847.

"6th. *Resolved*, That it is expedient that the Medical Profession in the United States should be governed by the same code of Medical Ethics, and that a Committee of seven be appointed to report a code for that purpose, at the meeting to be held in Philadelphia, on the first Wednesday in May, 1847."

Dr. O. S. Bartles, of New York, offered the following resolution, which after considerable discussion was referred to a committee of seven, by a vote of 58 to 23.

"*Resolved*, That the union of the business of teaching and licensing, in the same hands, is wrong in principle, and liable to great abuse in practice. Instead of conferring the right to license on medical colleges, and State and county medical societies, it should be restricted to one board, in such State, composed, in fair proportion, of representatives from the medical colleges, and the profession at large, and the pay for whose services, as examiners, should, in no degree, depend on the number licensed by them."

The Chairman announced the various committees on Dr. Davis' resolutions—as follows:—

"On the Organization of the National Medical Institution"—Drs. J. Watson, Stearns, Campbell, Stewart, Stille, Davis, Cogswell, Fenner.

"On the Address"—Drs. Knight, Ives, Dow, Sumner, McNaughton, Blatchford, Boswell, Baxley.

"On the Requirements for a Degree"—Drs. Haxhall, Cullen, Paterson (Va.), Norris, Flint, Perkins, Wing.

"On Preliminary Education"—Drs. Cowper, Bush, Thompson (Del.), March, Atlee, Brainard, Mead.

The closing business of the session, on Wednesday, as we gather from the New York papers, was as follows:—

Dr. Thompson's resolution of thanks to the Colleges, for the offer of their rooms for the Convention, was taken from the table and adopted. A member moved a resolution to call on the different medical societies, in the different States, to report the births, marriages and deaths in their several States. Carried.—A vote of thanks was then proposed to the officers of the Convention, for the manner in which they had discharged their duties. Carried unanimously.—A vote providing for the publication of the proceedings of the Convention, in pamphlet form, was then offered, and adopted.—A resolution was passed, providing for the arrangement of a system of nomenclature of diseases, with reference to the registration of deaths.—An invitation from Dr. Delafield (V. P.) to the members of the Convention, to visit him at his house to-morrow (Thursday) evening, was accepted, with thanks, and unanimously.—Dr. Bell (V. P.) moved that this Convention approve the designs and publication of the Sydenham (publishing) Society, in England. Adopted.—Dr. Cogswell offered a vote of thanks to the chairman for the manner in which he had discharged the duties of his office. Adopted.—Prof. Knight (P.) briefly returned his acknowledgments.—And the Convention then ad-journed, *sine die.*

